# Dynamic Tracking of Respiratory Rate and Quantitative Analysis of Heat Stress Response of Caged Broilers Based on Infrared Thermal Imaging Video Amplification Technology

**DOI:** 10.3390/ani16071115

**Published:** 2026-04-05

**Authors:** Caihua Lu, Jincheng He, Wenwan Zheng, Mengyao Wu, Sisi Hong, Fan Lin, Hongjie Su, Yuyun Gao

**Affiliations:** 1 College of Mechanical and Electrical Engineering, Fujian Agriculture and Forestry University, Fuzhou 350002, China; 52312047007@fafu.edu.cn (C.L.); 52412047019@fafu.edu.cn (W.Z.); 52512032001@fafu.edu.cn (M.W.); hss_1844165@163.com (S.H.); 3236129007@stu.fafu.edu.cn (H.S.); 2 Modern Agricultural Equipment Engineering Research Center of Fujian Universities, Fuzhou 350002, China; 3 State Key Laboratory of Agricultural Equipment Technology, Beijing 100083, China; linfan36@163.com; 4 Chinese Academy of Agricultural Mechanization Sciences, Beijing 100083, China; 5 College of Animal Sciences, Fujian Agriculture and Forestry University, Fuzhou 350002, China; gaoyuyun2000@163.com

**Keywords:** infrared thermal imaging technology, video magnification technology, caged broilers, respiratory rate, heat stress

## Abstract

Broiler respiratory rate (RR) in cage systems is a core physiological indicator of health and stress. However, real-time, non-invasive continuous RR monitoring is difficult in a high-density breeding environment, thereby limiting precise poultry health management. This study developed a feasible non-contact broiler RR measurement method to address this gap. The proposed method integrates infrared thermal imaging and phase-based video magnification (PBVM). Using cage-reared white-feathered broilers as subjects, we selected the thoracodorsal and tail regions as regions of interest (ROI), applied PBVM to amplify subtle respiratory-related body surface movements, and extracted RR features via the Fast Fourier Transform (FFT). Two validation experiments were conducted under controlled laboratory conditions. One was an RR dynamic monitoring experiment covering the entire life cycle (4 to 36 days), which analyzed video data of 198 individual quiet broilers. The other was a multi-gradient heat stress experiment with temperature increases of +2 °C, +4 °C, and +5 °C, and analyzed video data of 162 individual quiet broilers. The method achieved favorable measurement accuracy: in the whole-life-stage experiment, the mean absolute error (MAE) was 0.036 Hz, the mean absolute percentage error (MAPE) was 4.461%, and the coefficient of determination (*R*^2^) reached 0.961; in the heat stress experiment, the MAE was 0.042 Hz, the MAPE was 3.270%, and the *R*^2^ reached 0.928. Linear regression analysis confirmed that healthy broiler RR decreased linearly with increasing age, and verified that RR showed a stepwise response to thermal challenge with a positive correlation between RR increase and temperature increment, accompanied by growth stage specificity. This study provides a feasible non-invasive approach for broiler RR monitoring, offering preliminary reference data for early heat stress detection and sustainable poultry production.

## Simple Summary

In commercial poultry farming, monitoring the breathing of farmed broilers is crucial for tracking their health and stress levels. However, current methods cannot do this continuously, without touching the broilers, in crowded rearing environments. This gap limits proper broiler health care and responsible farm management. Our study aimed to explore a simple, non-contact way to track broiler breathing, using infrared thermal camera footage and technology that amplifies tiny body movements to pick up breathing signals. We validated this method across the whole life stage of broilers and under heat stress conditions induced by high temperature. The method gave accurate, reliable results matching manual counts, and we mapped how healthy broilers’ breathing changes as they grow, and how they respond to heat. This work provides farmers with a practical, animal-friendly tool to monitor broiler health. It helps identify stress and illness earlier, improve animal welfare, and support more sustainable poultry production for a safe, stable food supply.

## 1. Introduction

Respiratory rate (RR) monitoring is a crucial physiological indicator technology in poultry health management, especially for improving the accuracy of early health detection and management [1,2]. During the early stages of diseases or heat stress, poultry typically exhibit changes in respiratory rate, which usually occur before the clinically observable symptoms appear. Therefore, using non-invasive sensing technology to continuously monitor RR provides key data support for the early identification of poultry health conditions and heat stress risks [3].

Respiratory rate (RR) is widely recognized as a key physiological indicator of animal health and physiological status, and its variation is closely associated with environmental conditions, disease development, and husbandry management practices. When animals encounter heat stress, respiratory system diseases, or other external stressors, their RR often shows significant abnormalities first. Therefore, accurately measuring the RR is an essential prerequisite for conducting animal health assessments. Current approaches for measuring animal RR can be broadly classified into contact-based [4,5,6] and non-contact methods. Owing to their advantages of being non-invasive and suitable for continuous monitoring, non-contact techniques have been extensively applied in human clinical and healthcare monitoring [7], leading to the development of various methods based on acoustic, optical, and radio-frequency signals. However, the application of such non-contact respiratory monitoring techniques in animals—particularly in small poultry species such as broilers—remains limited, highlighting a clear need for further research.

Current research on the non-contact measurement of animal RR primarily focuses on the three technical approaches: Wi-Fi-based sensing [8], acoustic detection [9], and video processing technology [10,11,12]. However, the specific characteristics of caged broilers—small body size, weak respiratory signals, high stocking density, and frequent occlusions between individuals—impose higher demands on the applicability of non-contact monitoring techniques. The Wi-Fi-based sensing method is vulnerable to environmental interference, such as metal structures in cages, and often has limitations such as weak signal strength and low signal-to-noise ratio, making it difficult to be stably applied in densely caged broilers. The sound detection method is highly sensitive to background noise and broiler vocalizations. Specifically, the mechanical noise from continuous fan and feeder operation, alongside frequent broiler calls, limits the practical application of this method in high-density houses. In contrast, video processing technology, with its unique advantages such as intuitive visualization, non-contact operation, and high spatiotemporal resolution, can capture the weak physiological characteristics triggered by the body surface breathing of broilers through image sequences in real time. In particular, infrared thermal imaging technology can effectively avoid the interference of non-uniform illumination in the cage and color differences in feathers in visible light images, directly capturing the body surface fluctuations triggered by breathing. Subsequently, combined with motion amplification technology, it enhances the detection ability of weak physiological signals and shows stronger applicability in complex breeding scenarios. Therefore, video processing technology has gradually become an important research direction in the field of non-contact respiratory monitoring of animals.

Video processing technology for RR detection in livestock and poultry can be classified into two categories: visible light RGB imaging and infrared thermal imaging. Both approaches rely on computer vision–based analysis of video sequences to extract respiration-related features and estimate RR. Among them, visible light RGB technology captures cyclical fluctuation signals around the chest, abdomen, or nostrils of livestock and poultry, parses body movement trajectories by relying on optical flow algorithms or key point detection technology, and combines time-domain signal analysis methods to extract the respiratory cycle. For example, Wu et al. [13] combined convolutional neural networks (CNN) with bidirectional long short-term memory networks (Bi-LSTM) to process RGB videos of dairy cows. By analyzing the motion characteristics of the chest and abdomen, they achieved RR detection with an accuracy of 93.56% on the test set.

Infrared thermal imaging technology, however, monitors thermal radiation differences in the nasal exhaled airflow or periodic temperature fluctuations on the body surface of the chest and abdomen, and calculates RR based on temperature field analysis algorithms. Stewart et al. [14] monitored nostril temperature changes in dairy cows using a ThermaCam S60 thermal camera (FLIR Systems, Inc., Wilsonville, OR, USA), reporting an error of only 0.83 ± 0.57 times per minute compared with manual counting. Similarly, Kim et al. [15] utilized a FLIR One Pro thermal imaging camera, and the determination coefficient (*R*^2^) between the RR measured by the average nasal temperature method and the manual observation value was 0.91. The above studies show that video processing technologies based on visible RGB or infrared thermal imaging have achieved good results in the monitoring of animals with large body sizes, significant respiratory movements, or temperature changes, such as dairy cows.

To address the challenge of detecting weak physiological signals, video magnification techniques [16] have been gradually introduced into the field of animal physiological parameter measurement. This technique was initially proposed by Wu et al. [17] from the Massachusetts Institute of Technology, and the classical technical scheme is Eulerian Video Magnification (EVM). Subsequent researchers have proposed a series of improved schemes for EVM [18,19], and some scholars have also conducted research and summarized it [20]. Its core advantage is its ability to significantly amplify subtle color or motion changes in videos, which provides technical support for the visual analysis of subtle physiological signals. In the practical application of animal physiological monitoring, Lauridsen et al. [21] successfully used EVM to detect physiological signals such as heart rate and RR in both laboratory and field animals, realizing the localization and recording of latent rhythmic signals. Wu et al. [22] proposed a method for detecting the cattle RR by combining the DeepLabV3+ semantic segmentation model, Phase-based Video Magnification (PBVM) algorithm, and optical flow algorithm, with an average accuracy of 93.04%. For broilers, Wang et al. [23] adopted an improved single-channel EVM algorithm to estimate RR by analyzing abdominal fluctuation characteristics, with an average accuracy of 92.19%. Caroline et al. [24] integrated infrared thermal imaging with EVM to measure the RR and heart rate of 52 species, which showed high accuracy in mammals. However, the technique’s performance in reptiles and birds is limited due to skin and feather properties and motion artifacts, indicating that further optimization is required for these animal groups.

Heat stress is the primary environmental stressor in broiler production, severely compromising the health and production performance of broilers [25]. Previous studies have extensively explored heat stress, including the mechanism by which heat stress induces lung damage in broilers [26], as well as the analysis of the influencing factors of heat stress and their multiple detrimental effects on broiler production [27]. When the environmental temperature exceeds the thermal comfort zone, the RR of broilers increases significantly with a positive correlation [28]. Heat stress induces oxidative stress and metabolic disturbances by activating the hypothalamic–pituitary–adrenal (HPA) axis, and elevated RR is the core physiological compensatory response for broilers to maintain body temperature homeostasis through evaporative heat dissipation. This mechanism exists in broilers of different ages, but the intensity of the RR response varies [29].

Previous studies have confirmed that fast-growing broilers, due to their higher metabolic rate and relatively underdeveloped cardiovascular and respiratory systems, have a greater increase in RR under heat stress than conventional strains and are more sensitive to temperature changes [28,30]. The increase in RR is often accompanied by characteristic behaviors such as open-mouth breathing. Although existing studies have developed tools for detecting open-mouth breathing in cage-reared broilers, based on static images, behaviors such as feeding and vocalization can be mistakenly identified as open-mouth breathing, making it impossible to distinguish short-term behaviors from continuous open-mouth breathing, and without considering environmental temperature, it is difficult to distinguish physiological from pathological open-mouth breathing [31]. Currently, most non-contact measurement studies of broiler breathing are limited to laboratory scenarios of individual broilers and have not undergone method validation under heat stress conditions [23], or have explored the impact of heat stress on broilers by calculating the broiler activity index BAI through image processing [32]. There is still a lack of non-contact, continuous, real-time monitoring of the dynamic changes in RR in cage-reared broilers. Given the current research status, this study focuses on the cage-reared white-feathered broilers, a typical fast-growing strain, aiming to establish a non-contact continuous monitoring method applicable to cage-rearing conditions.

Although the studies mentioned above provide important references for non-contact RR measurement in animals, an analysis of practical broiler production needs and existing research results reveals three core research limitations and areas for improvement:

1. Limited technological adaptability and measurement solutions for broilers require further development. Current research on RR measurement has primarily focused on large animals such as pigs, sheep, and dairy cows. Studies on broilers still rely mainly on manual observation or respiratory sound monitoring, which have the limitations of high labor costs and low measurement accuracy. Broilers are small in size with weak respiratory signals. In cage-rearing environments with high density and serious mutual occlusion between individuals, traditional contact devices are prone to inducing stress responses, leading to measurement deviations. Non-contact video processing technology, however, has room for optimization in broiler scenarios and has not yet formed a widely recognized, mature measurement solution. Although manual observation is simple to perform, it is easily affected by the observer’s subjective judgment and cannot achieve continuous dynamic monitoring [33]. Video image processing technology is an effective non-contact method for measuring RR in poultry because it can capture subtle physiological movements, but research on its adaptability to high-density cage-rearing scenarios requires further investigation.
2. Whole-life-stage dynamic monitoring data and standardized reference systems need improvement. Most existing studies on broiler RR focus on “single-time period” monitoring or targeted measurement under specific conditions, and systematic studies on the dynamic change patterns of RR throughout the whole life stage are relatively limited. Although Nascimento et al. [28] completed weekly RR measurements of broilers aged 0–6 weeks under controlled temperature conditions and confirmed that RR varies significantly with age, such studies are limited in number. Their core goal is comparative analysis under heat stress rather than establishing a whole-life-stage standard physiological data model. A systematic database or standardized reference value system covering the complete growth stage of broilers has not been fully established, which fails to provide adequate data support for judging respiratory abnormalities at different growth stages. The baseline RR of broilers shows a downward trend with increasing age, which is closely related to the decrease in metabolic rate and the gradual improvement of thermoregulatory capacity [34]. However, existing research on continuous tracking and quantitative modeling of this dynamic pattern is insufficient.
3. Insufficient adaptability to heat stress scenarios and the need for further research on dynamic change rules. Although existing studies have clarified the correlation between RR and broiler heat stress, two areas for improvement remain when combined with actual farming scenarios. First, the abnormal response characteristics of RR under heat stress have not been fully verified by video measurement technology, and the applicability of the video magnification measurement method proposed in this study in such scenarios remains to be clarified. Second, the exploration of dynamic change patterns of broiler RR under different heat stress intensities is not yet comprehensive, which cannot adequately support the early warning and precise regulation of heat stress. There is a significant corresponding relationship between heat stress intensity and RR. Yin et al. [35] showed that the RR of broilers under a 32 °C heat stress environment is significantly higher than that under an optimal 23 °C environment. However, most existing studies focus on a single temperature gradient, and quantitative analysis of dynamic response rules under multi-gradient temperature increases is relatively limited.

To address the research gaps outlined above, this study focuses on caged white-feathered broilers and aims to explore a non-contact RR measurement method based on video magnification technology. The main research content is as follows. Firstly, this study explores a non-contact method for measuring RR in broilers by using video magnification technology to enlarge the subtle respiratory fluctuations in the thoracodorsal and tail regions, attempting to solve the problem of weak respiratory signals and susceptibility to interference in the cage environment. Secondly, it conducts dynamic monitoring of the RR throughout the growth stage of broilers, analyzes the changing patterns of RR, and establishes a preliminary reference system for the entire life cycle. Thirdly, under graded heat stress conditions, the applicability and accuracy of the proposed method are initially verified, and the dynamic response patterns of the broiler RR to different heat stress intensities are investigated. This helps to further quantify the correlation between increased RR and age-related thermal regulation sensitivity [36].

These research results provide a promising technical approach for heat stress monitoring of cage-reared broilers, and offer preliminary data and technical references for health management and heat stress control. They have certain theoretical and practical significance for promoting the development of precision animal husbandry, maintaining production efficiency, and protecting animal welfare.

## 2. Materials and Methods

### 2.1. Test Site and Test Animals

**Figure 1:**
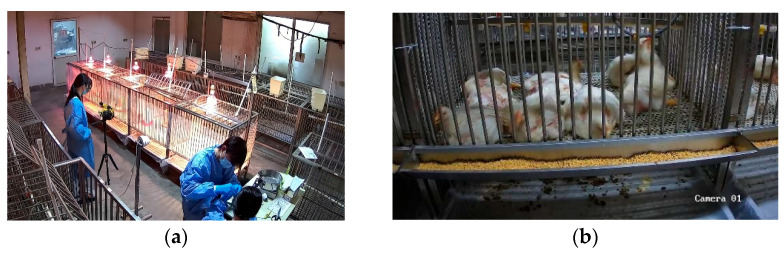
Broiler Farming Environment. (**a**) The experimental site for data collection; (**b**) The cage-raising unit for white-feathered broilers.

This study was conducted from September to October 2024 at the Poultry Incubation Laboratory of the Experimental Center, College of Animal Science, Jinshan Campus, Fujian Agriculture and Forestry University (FAFU). On 18 September 2024, 40 healthy 1-day-old white-feathered broilers (half male and half female) were randomly selected from the Chongren No. 2 Hatchery of Shengnong Group Co., Ltd., Nanping, China. Referring to the sample sizes used in similar non-contact respiratory monitoring studies (n = 30) [23], the sample size in this study was considered sufficient to ensure statistical reliability. The broilers were randomly assigned to five cages (0.9 × 0.75 × 0.6 m, length × width × height) for rearing, with eight broilers per cage (Figure 1). The housing unit consisted of eight broilers per cage, whereas the analytical unit in the subsequent data analysis was individual broilers in a quiet state, with each analytical sample derived from randomly selected independent individuals.

Prior to the experiment, all broilers underwent a 3-day acclimation period. During this period, the rearing conditions were consistent with those in the formal experiment. The broilers were fed with standardized commercial feed provided by Shengnong Group; feeding frequency was adjusted according to age following the Shengnong husbandry protocol, with water provided ad libitum; environmental temperature and humidity were maintained within the specified ranges using fans, air conditioners, and sprinkler systems (see Section 2.2 for specific parameters). During the acclimation period, only routine observations were conducted, and no data were collected to ensure that the broilers could adapt naturally to the experimental environment.

In this study, 40 broilers were tracked through long-term repeated observation, and the data collection covered the complete growth stage from 4 to 36 days of age. During the rearing period, one broiler was eliminated at 8 days of age due to weakness, abnormal feces, and poor condition. The remaining 39 broilers remained in good health throughout the entire experiment; no other deaths or disease events occurred, and no additional individuals were removed.

### 2.2. Test Environment Control

**Figure 2:**
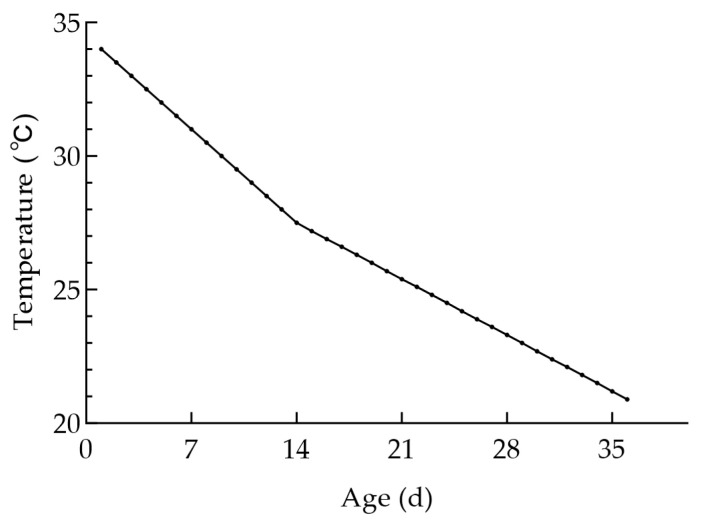
Optimal temperature for raising 1–36 days old broilers.

Throughout the entire rearing period, ambient temperature, relative humidity, and illumination intensity were dynamically adjusted according to broiler age to minimize heat stress and environmental interference:

1. Temperature regulation: During the early stage, temperature was decreased by 0.5 °C per day, and during the later stage by 2 °C per week (approximately 0.3 °C per day on average) [37,38]. Actual recorded values showed that the temperature was maintained within ±0.7 °C of the target during the rearing period. In the heat stress experiment, an intelligent temperature-controlled heating lamp was used to regulate ambient temperature, with a control accuracy of ±0.4 °C. Temperature was monitored jointly using hygrothermographs and temperature-humidity sensors. When the temperature deviated from the set range, it was regulated using air conditioners, ventilation systems, or sprinkler equipment. The detailed temperature profile is shown in Figure 2.
2. Humidity regulation: Relative humidity (RH) was maintained between 50% and 70%. A dynamic pattern of higher RH in early stages followed by gradual reduction was applied as the broilers matured [37,38]. Actual recorded values showed that humidity was maintained within ±5% RH of the target. Humidity was monitored jointly using hygrothermographs and temperature-humidity sensors. When the humidity was too low, the sprinkler system was activated for humidification. When the humidity was too high, the ventilation system was activated for dehumidification.
3. Illumination intensity: This was maintained between 30 and 40 lux throughout the experiment [39,40]. Illumination intensity was monitored using illuminance sensors, and LED light strips were used for lighting. Actual recorded values remained stable within the set range.

### 2.3. Experimental Design

#### 2.3.1. Data Collection During the Conventional Rearing Period

The experiment lasted for 33 days, covering broilers from 4 to 36 days of age. Broilers were classified into three growth stages according to age: the brooding stage (1–10 days), the growing stage (11–25 days), and the fattening stage (26–36 days). This experiment employed a repeated-measures design with longitudinal continuous tracking of the same 40 broilers. Every afternoon, broilers in a quiet state were prioritized for infrared thermal video recording. A quiet state was defined as: broilers in standing or lying posture, without obvious active movements such as running, jumping, or vigorous wing flapping. Data collection was carried out once every afternoon, during which a total of 100 one-minute video clips were collected. These 100 clips were obtained from the five cages, with 20 clips acquired per cage. Recording was performed in a loop, starting from cage 1 to cage 5 in sequence, and the process was repeated after each round was completed. Broilers within the same cage were repeatedly recorded at different time points on the same day to capture respiratory signals under various states. For subsequent analysis, six non-repeated broilers in a quiet state were randomly selected each day as independent analytical samples, with each broiler included only once to ensure sample independence. A total of 3300 video clips under standard rearing conditions were collected throughout the experiment.

The video screening criteria: Collected videos were manually screened, and clips were excluded if they met any of the following criteria: (1) unstable footage due to camera shake; (2) broilers in an obviously active state (running, jumping, vigorous wing-flapping); (3) blurred images or insufficient contrast. After screening, 2870 valid video clips were retained for subsequent analysis.

#### 2.3.2. Data Collection During Heat Stress Experiment

Heat stress experiments were carried out for three consecutive days in the late phase of each growth stage: the brooding stage (7–9 days), the growing stage (18–20 days), and the fattening stage (30–32 days), accounting for a total of nine experimental days. In each experiment, the videos of broilers were first recorded under optimal temperature, as described in Section 2.3.1. The environmental temperature was then increased sequentially by 2 °C, 4 °C, and 5 °C, with data collection performed at each temperature level. The temperature gradient setting was based on the results of the preliminary experiments. When the temperature rose by more than 5 °C, broilers exhibited irreversible heat stress reactions such as open-mouth breathing, rapid breathing, a significant decrease in feed intake, and listlessness, which affected the animal’s health and the subsequent experiments. Therefore, in this study, +2 °C, +4 °C, and +5 °C were selected as the heat stress gradients to induce heat stress responses while ensuring animal welfare.

The data collection process for heat stress is as follows. At each temperature gradient, the broilers were exposed to the temperature environment for 30 min to achieve thermal equilibrium, and then video recording was conducted. The recording was carried out in a cyclic manner, with each temperature gradient being shot in sequence from cage 1 to cage 5, for a total of 3 cycles. Each cage was shot for 3 one-minute videos in sequence, totaling 15 videos. After completing the video data collection for one temperature gradient, the temperature setting and data collection for the next temperature gradient were carried out without a recovery period to simulate the continuous temperature increase scenario in actual breeding. After the +5 °C temperature gradient data collection was completed, the environmental temperature gradually returned to the normal breeding temperature, and the broilers rested for 30 min to ensure their physiological state returned to normal. 15 one-minute video clips are collected for each temperature gradient, and 3 temperature gradients were collected each experimental day. The entire heat stress experiment collected a total of 405 video clips. According to the video screening criteria mentioned in Section 2.3.1, after screening these videos, a total of 355 valid video clips were obtained and were used for subsequent analysis.

### 2.4. Data Acquisition Equipment and Parameters

#### 2.4.1. Infrared Thermal Imaging Equipment

**Figure 3:**
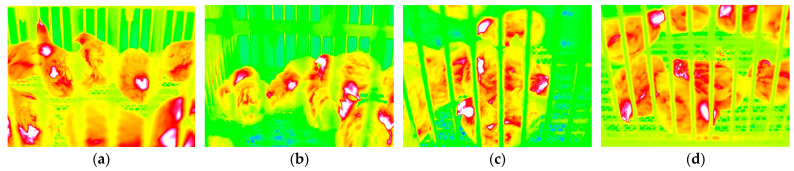
Infrared thermal video frames of white-feathered broilers. (**a**) Frame captured from Video 1; (**b**) Frame captured from Video 2; (**c**) Frame captured from Video 3; (**d**) Frame captured from Video 4.

Infrared videos of broilers were recorded using a TiX660 infrared thermal imager (Fluke Corp., Everett, WA, USA). The device parameters are listed in Table 1, and the captured infrared thermal video frames are presented in Figure 3. The shooting angle was positioned directly in front of the broiler cage, as shown in Figure 1a.

#### 2.4.2. Data Processing Hardware and Software Configuration

The hardware and software configurations employed for video data processing and RR calculation are listed in Table 2.

### 2.5. True Respiratory Rate Acquisition

True RR values were obtained through manual observation for comparison with the experimental measurement results [23]. The principle is as follows: The respiratory movements of broilers are driven by the contraction and relaxation of respiratory muscles, which in turn drive the expansion and retraction of the thoracic cage. These movements are transmitted to the body surface through tissues such as the chest and back muscles, skin, and feathers, resulting in regular undulations in the chest and back regions. Meanwhile, during broiler respiration, the air sacs expand and contract synchronously with the thoracic cage movements. Since the air sacs extend to the vicinity of the tail, their movements can drive the tail to produce subtle undulations consistent with the respiratory rhythm.

Based on the aforementioned respiratory physiology and under the guidance of experienced poultry farming experts, three trained observers independently performed manual counts on video segments of broilers in a quiet state. The observers used the rising and falling of the thoracodorsal region and tail as indicators of respiration. The observation window was defined as a 10 s continuous video segment, which was identical to the segment used for RR calculation based on video magnification technology. A complete respiratory cycle, in which the thoracodorsal region and tail exhibited a full undulation, was counted as 1 breath, whereas a partial cycle that did not complete a full wave was counted as 0.5 breath. Each observer performed three independent counts on the 10 s video segment for each broiler (at different time points), and the average of these three counts was taken as the observer’s count. Subsequently, the average of the three observers’ counts was calculated to obtain the number of breaths in 10 s, which was then divided by 10 to obtain the RR in Hz (breaths per second). After the examination, the inter-observer reliability was evaluated at 0.93 by the intraclass correlation coefficient (ICC), and the ICC values of the reliability of each of the three observers were 0.95, 0.97, and 0.96, respectively, indicating that the manual counting had good consistency.

### 2.6. Measurement of Respiratory Rate

#### 2.6.1. Overall Measurement Process

**Figure 4:**
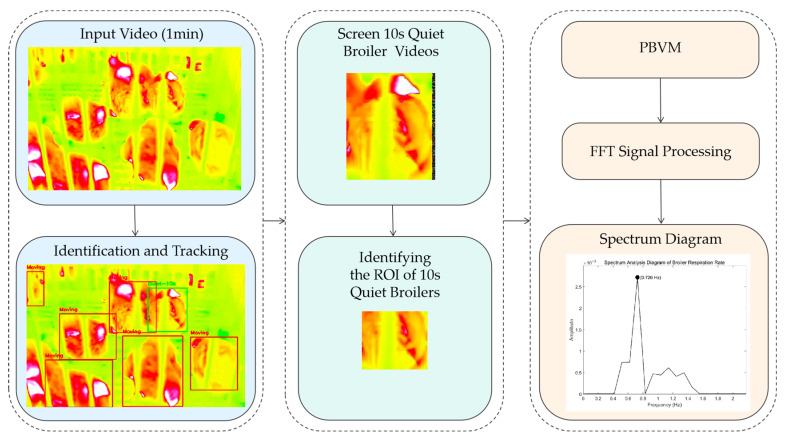
Flowchart for obtaining the RR of broilers.

This study utilized infrared video technology to measure the RR in broilers. Throughout the measurement process, the broilers were kept in a quiet state to minimize motion artifacts and ensure data accuracy and reliability. The complete measurement workflow was divided into three core modules, as illustrated in Figure 4.

1. Input Tracking Module: The system inputted 1 min infrared thermal video clips. An improved Real-Time Detection Transformer (RT-DETR) deep learning model, which had been fully trained on a dataset containing broilers of different ages and postures, was used to identify and locate individual broilers. Subsequently, dense optical flow was applied to continuously track the identified broilers and obtain their motion trajectories.
2. Screening and Localization Module: Based on the tracking results, the motion amplitude of the broilers within a 10 s time window was calculated using the dense optical flow method, and the motion amplitude threshold was set at 1.5 pixels. Broilers with no obvious body movement within this window (i.e., “quiet state” broilers) were selected. Subsequently, the trained improved RT-DETR model was used to automatically locate the regions of interest (ROIs) related to respiratory movements.
3. Signal Analysis Module: Video data within the ROIs were processed using phase-based video magnification (PBVM) technology to enhance subtle physiological signals corresponding to respiratory movements. The amplified signals were then processed using fast Fourier transform (FFT) spectral analysis, enabling the estimation of RR.

Due to space limitations, detailed preprocessing procedures are described in a separate publication.

#### 2.6.2. Preprocessing: Acquiring Quiet Broilers and Their ROIs

The primary objective of preprocessing was to identify quiet broilers that satisfied the measurement requirements and to localize regions of interest (ROIs) associated with respiratory movements. This provided a foundation for subsequent signal amplification and frequency calculation. The key technical considerations are described below.

1. Selection criteria for quiet broilers: This study referred to the research conclusions of Leen Yassin Kassab et al. [41] on the influence of video window length on the performance of video magnification technology. When the head movement of the target object is minimal, a window length exceeding 10 s reduces the detection performance of the video magnification algorithm for subtle and transient changes in heart rate. Considering the weakness and transience of broiler RR signals, this study adopted 10 s as the time threshold for judging the quiet state. Broilers that remained stationary within 10 s, with no obvious body swaying or displacement, were selected to ensure the detection efficiency of subsequent algorithms for subtle respiratory movements.
2. Physiological basis for ROI localization: Combined with the respiratory physiological characteristics of broilers, their breathing process is mainly realized by the regular undulation of the thoracic cage and the contraction and expansion of air sacs. The chest and back exhibit synchronous undulations with the movement of the thoracic cage, while the tail produces subtle displacement due to the contraction and expansion of air sacs. The movement rhythms of both are consistent with the RR. Based on this physiological mechanism, this study jointly located the thoracodorsal region and the tail as ROIs for RR measurement, ensuring that the collected signals directly reflect the respiratory physiological state of broilers.

#### 2.6.3. Video Magnification Technology

To capture the subtle body surface motion signals induced by respiratory activity in broilers, this study compared the characteristics of two video magnification techniques and selected the more suitable approach for signal enhancement. Below, the fundamental principles of Eulerian Video Magnification (EVM) are briefly elaborated. Subsequently, the implementation process and core mechanisms of Phase-Based Video Magnification (PBVM) are described in detail, clarifying the basis for selecting the technical scheme for this study.

EVM technology [17] is a commonly used method for enhancing subtle motions in videos. Its core workflow employs multi-stage signal processing to amplify motion variations that are difficult to perceive with the naked eye. First, video frames are transformed from the original color space into a space more suitable for signal processing, laying a foundation for subsequent operations. Then, image pyramids are used to decompose the video sequence into multiple spatial resolution scales. Common techniques include Laplacian pyramids, steerable pyramids, and Gaussian pyramids, which can be flexibly selected according to the characteristics of the video data. Next, frequency domain filtering is performed on the decomposed images of each scale to screen out the frequency bands of interest containing respiratory motion and to eliminate background noise and irrelevant signal interference. Then, the filtered target signals are multiplied by a specific amplification factor (*α*) to enhance the detectability of subtle motions through linear amplification, and the amplified signals are superimposed on the original signals. Finally, inverse pyramid reconstruction is performed on the processed multi-scale images to restore them to complete magnified images and to reconstruct video sequences containing enhanced subtle motions.

In the scenario of respiratory signal detection in broilers, EVM technology has certain limitations. Its magnification factor is limited, and it is prone to noise distortion, which makes it difficult to meet the enhancement needs of subtle respiratory motions. Therefore, this study adopted PBVM technology [18] for signal processing. This technology exhibits superior performance in enhancing weak regular motions, and its core implementation process and principles are as follows.

1. Spatial Decomposition: A complex-valued steerable pyramid was used to perform multi-scale and multi-orientation decomposition of the 10 s ROI video. This pyramid captures the local phase and amplitude information of images via complex filters. It can not only analyze the directional characteristics of images but also characterize the directionality of local structures such as edges and textures, realizing efficient linear decomposition of video frames into scale and direction sub-bands. In this study, the number of pyramid levels was adaptively determined based on image size, ranging from 1 to 4 levels. The number of orientations was fixed at 8.
2. Temporal Filtering: An ideal filter was selected to perform frequency domain filtering on the decomposed images of each scale. The filtering frequency band was restricted to the range of broiler RR, i.e., 0.3 Hz to 3.0 Hz, thereby preserving respiration-related motion signals. The absolute bandwidth of the temporal filter was 2.7 Hz.
3. Linear Amplification: The filtered phase signals were multiplied by the amplification factor *α* to achieve linear amplification of subtle respiration-related motions. In this study, the amplification factor *α* was fixed at 40. Unlike EVM technology, this study did not require subsequent video reconstruction steps. The amplified motion signals were directly extracted for subsequent frequency analysis, avoiding potential signal distortion that may occur during the reconstruction process.

The core principle of PBVM is based on the Fourier series decomposition of image displacement–intensity distributions, through which motion magnification is achieved by phase modification. The fundamental idea is to first establish a one-dimensional motion model of image intensity and then progressively perform signal decomposition, filtering, and amplification. Specifically, PBVM considers a one-dimensional image intensity distribution f undergoing global translation over time t. That is, given a displacement function δ(t), the displaced intensity distribution can be expressed as f(x+δ(t)). By introducing an amplification factor α, a motion-amplified sequence with modified displacement characteristics is constructed as f(x+(1+α)δ(t)). On this basis, the displacement–intensity distribution of the image can be represented as a superposition of complex sinusoidal components at different frequencies through Fourier series decomposition. The corresponding mathematical expression is given as follows:

(1) $$f(x+\delta (t))=\sum_{\omega =-\infty }^{\infty }A_{\omega }\;\times \;e^{i\omega (x+\delta (t))}$$

Each complex sinusoidal component corresponds to a specific frequency band ω. Further analysis of Equation (4) indicates that the component associated with frequency ω can be expressed independently as Equation (2), as follows:

(2) $$S_{\omega }(x,t)=A_{\omega }{\;\times \;e}^{i\omega (x+\delta (t))}$$

Since $S_{\omega }(x,t)$ is essentially a sinusoidal wave, its phase term ω(x+δ(t)) encodes critical motion information. This property provides the theoretical basis for achieving motion manipulation via phase modification.

To extract the motion signal at a specific frequency-spatial scale, a DC-balanced filter is applied to perform time-domain filtering on the phase term ω(x+δ(t)). Assuming that only the DC component ωx is removed during filtering, the resulting phase signal contains solely the motion-related information, as expressed in Equation (3):

(3) $$B_{\omega }(x,t)=\omega \times \delta (t)$$

By multiplying the bandpass-filtered phase signal $B_{\omega }(x,t)$ by the amplification factor α and subsequently superimposing the result onto the phase of the original sub-band $S_{\omega }(x,t)$, a sub-band signal exhibiting motion amplification is obtained. The corresponding expression is as follows:

(4) $${\hat{S}}_{\omega }(x,t)=S_{\omega }(x,t)\times e^{i\alpha B_{\omega }}=A_{\omega }\times e^{i\omega (x+(1+\alpha )\delta (t))}$$

Equation (4) shows that ${\hat{S}}_{\omega }(x,t)$ remains a complex sine wave, with its motion amplitude relative to the original input signal precisely amplified by a factor of (1+α), thereby achieving the desired motion enhancement. In the context of detecting respiratory signals in broilers, this process amplifies the subtle surface movements induced by respiration. The final motion-amplified video sequence f(x+(1+α)δ(t)) is obtained by summing all amplified sub-band signals, providing a reliable signal basis for subsequent RR estimation.

#### 2.6.4. Respiratory Rate Estimation Based on Spectral Analysis

After obtaining the motion-amplified signal sequence, it was transformed into the frequency domain using the Fast Fourier Transform (FFT). The FFT is an efficient computational algorithm for the Discrete Fourier Transform (DFT), enabling the mapping of a time-domain signal $f\left[n\right]$ to the frequency-domain representation $F\left[k\right]$. This transformation facilitates the quantitative analysis of the signal’s frequency components. The process can be expressed as follows:

(5) $$F\left[k\right]=\sum_{n=0}^{N-1}f\left[n\right]\times e^{-i\frac{2\pi }{N}kn}$$

where *N* denotes the signal length (number of samples), *k* represents the frequency index ranging from 0 to *N* − 1, and $\;F\left[k\right]$ is a complex number representing the amplitude and phase of the signal at frequency *k*.

The frequency-domain signal obtained via the FFT is a complex sequence that requires further spectral analysis to extract RR-related frequency characteristics. Specifically, the magnitude spectrum of the sequence is calculated to represent the signal energy corresponding to each frequency component. Since the breathing movement of broilers is periodic, the corresponding frequency components will form energy peaks in the amplitude spectrum; and the body surface movement energy caused by breathing is relatively concentrated in a specific frequency range.

This study adopted the peak validity determination rule: only the peaks located within the effective respiratory frequency band of 0.3 Hz to 3.0 Hz were retained. When multiple peaks appeared in the spectrum, the peak with the largest global amplitude within the 0.3 Hz to 3.0 Hz range was selected as the true RR, and the remaining peaks were all regarded as interference and eliminated. Therefore, the frequency corresponding to the highest amplitude peak in the amplitude spectrum is the main frequency of the broiler’s respiratory movement.

### 2.7. Evaluation Indicators

To systematically evaluate the accuracy of the broiler RR measurement algorithm in this experiment, core indicators were selected from two dimensions: statistical analysis and predictive performance.

#### 2.7.1. Basic Characteristics of Statistical Analysis

In this study, the standard deviation (SD) and the standard error of the mean (SEM) were used to describe data dispersion and the reliability of the mean estimation, while the Pearson correlation coefficient (*r*) was used to quantify the linear correlation strength between the algorithm-derived values and manual counts. The calculation formulas are as follows:

1. Standard Deviation (SD): This describes the absolute dispersion of measurement data, reflecting the extent to which individual measurements deviate from the mean. A smaller SD indicates a higher degree of data centralization. The formulas are provided in Equations (6) and (7).
  (6) $$\sigma =\sqrt{\frac{\sum_{i=1}^{n}{\left(y_{i}-\bar{y}\right)}^{2}}{n}}$$
  (7) $$SD=\sqrt[{}]{\frac{\sum_{i=1}^{n}{\left(y_{i}-\bar{y}\right)}^{2}}{n-1}}$$
2. Standard Error of the Mean (SEM): This measures the error and reliability of the sample mean in estimating the population mean. A smaller SEM indicates higher estimation accuracy. The calculation formula is shown in Equation (8).
  (8) $$SEM=\frac{SD}{\sqrt{n}}$$
3. Pearson Correlation Coefficient (*r*): This quantifies the linear correlation strength between the algorithm-derived values and the manual counts, ranging from −1 to 1, with a larger absolute value indicates a stronger linear correlation. The calculation formula is shown in Equation (9).
  (9) $$r=\frac{\sum_{i=1}^{n}\left(y_{i}-\bar{y}\right)\left(x_{i}-\bar{x}\right)}{\sqrt{\sum_{i=1}^{n}{\left(y_{i}-\bar{y}\right)}^{2}}\sqrt{\sum_{i=1}^{n}{\left(x_{i}-\bar{x}\right)}^{2}}}$$
  where n represents the sample size, $y_{i}$ represents the actual artificial count value of the *i*-th instance, $\bar{y}$ is the average value of the artificial count values, $x_{i}$ represents the algorithm measurement value of the *i*-th time, and $\bar{x}$ is the average value of the algorithm measurement values.

#### 2.7.2. Evaluation Metrics for Prediction Accuracy

The mean absolute error (MAE), the mean absolute percentage error (MAPE), the root mean square error (RMSE), and the coefficient of determination (*R*^2^) were used to evaluate the measurement accuracy of the algorithm. The calculation formulas are as follows:

1. Mean Absolute Error (MAE): This reflects the average magnitude of deviation between the algorithm-derived values and the manual counts. A smaller MAE indicates lower individual measurement deviation. The calculation formula is shown in Equation (10).
  (10) $$MAE=\frac{1}{n}\sum_{i=1}^{n}\left|y_{i}-x_{i}\right|$$
2. Mean Absolute Percentage Error (MAPE): This quantifies the relative error magnitude relative with respect to the true value, providing an intuitive measure of relative deviation as a percentage. The calculation formula is shown in Equation (11).
  (11) $$MAPE=\frac{1}{n}\sum_{i=1}^{n}\left|\frac{y_{i}-x_{i}}{y_{i}}\right|\times 100\%$$
3. Root Mean Square Error (RMSE): This quantifies the overall deviation between the algorithm-derived values and the manual counts, with greater sensitivity to large errors. A smaller RMSE indicates higher overall measurement accuracy. The calculation formula is shown in Equation (12).
  (12) $$RMSE=\sqrt{\frac{1}{n}\sum_{i=1}^{n}{\left(y_{i}-x_{i}\right)}^{2}}$$
4. Coefficient of Determination (*R*^2^): This quantifies the extent to which the algorithm-derived values explain the variation in the manual counts. The $R^{2}$ in this study represents the linear fitting degree between the manual reference values ($y_{i}$) and the algorithm-measured values ($x_{i}$) relative to the identity line. The calculation formula is shown in Equation (13).
  (13) $$R^{2}=1-\frac{\sum_{i=1}^{n}{\left(y_{i}-x_{i}\right)}^{2}}{\sum_{i=1}^{n}{\left(y_{i}-\bar{y}\right)}^{2}}$$
  where n represents the sample size, $y_{i}$ represents the actual manual count value of the *i*-th instance, $\bar{y}$ is the average value of the manual count values, $x_{i}$ represents the algorithm measurement value of the *i*-th instance, and $\bar{x}$ is the average value of the algorithm measurement values.

All statistical analyses and plotting in this study were performed using GraphPad Prism 10.1.2 software (GraphPad Software, San Diego, CA, USA). Differences between groups were tested using one-way analysis of variance (ANOVA), followed by Tukey’s post hoc test for pairwise comparisons. Trend fitting was performed using linear regression analysis, with *R*^2^ used to evaluate the goodness of fit.

## 3. Results

### 3.1. RR Measurement Results

**Figure 5:**
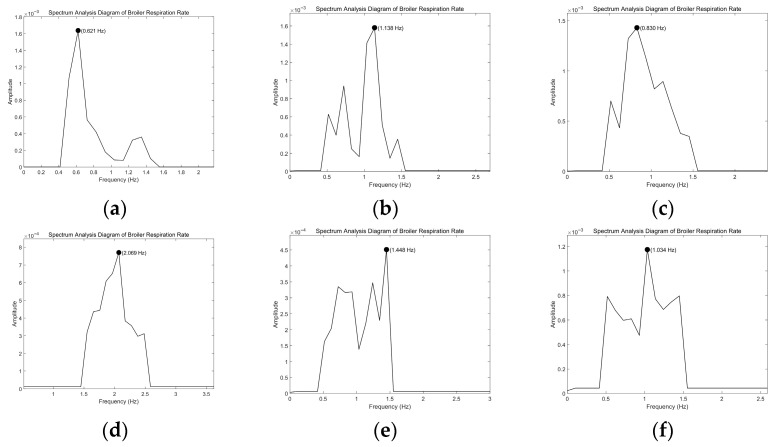
Spectral diagram of broiler RR. (**a**) Brooding stage (optimal temperature). (**b**) Growing stage (optimal temperature). (**c**) Fattening stage (optimal temperature). (**d**) Growing stage (temperature increase of 2 °C). (**e**) Growing stage (temperature increase of 4 °C). (**f**) Growing stage (temperature increase of 5 °C).

Based on the RR measurement method for broilers described in Section 2.6, this study successfully obtained the RR data of broilers across all growth stages and periods, as well as under different levels of heat stress. The representative measurement results are collectively presented in a composite spectrum plot (Figure 5). This composite diagram contains six subgraphs corresponding to the brooding stage, growing stage, and fattening stage, along with the RR spectral characteristics of growing broilers under heat stress with temperature increases of 2 °C, 4 °C, and 5 °C (taking growing broilers as an example). In all subgraphs, the peak of the primary RR with the highest amplitude can be clearly identified. This confirms that this measurement method can effectively extract the RR signals of broilers under different physiological states and environmental conditions. Detailed quantitative analysis of the measurement method accuracy is presented in Section 3.2.

### 3.2. Analysis of Monitoring Method Accuracy

#### 3.2.1. Experiment on RR of Broilers Throughout the Whole Life Stage

The whole-life-stage experimental data were obtained from 6 randomly selected, mutually independent healthy broilers per day of age, ranging from 4 to 36 days of age. A total of 198 observations were finally acquired. RR was measured by two methods: manual counting and algorithm-based measurement.

**Figure 6:**
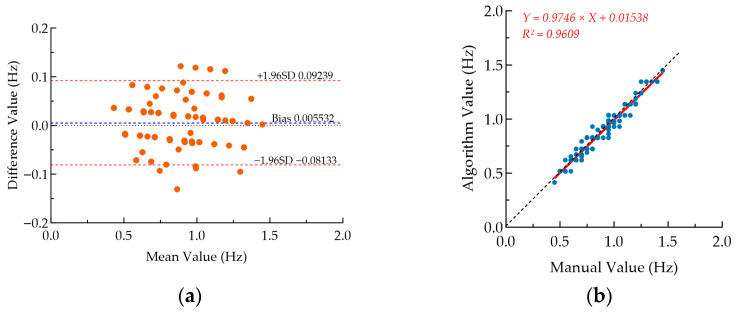
Consistency and correlation analysis between manual measurements and algorithm calculations across the whole life stage. (**a**) Bland–Altman plot of manual versus algorithm values. The blue dashed line represents the mean bias (Bias = 0.005532 Hz), while the red dashed line denotes the 95% consistency limits corresponding to ±1.96 standard deviations (−0.08133 Hz to 0.09239 Hz). (**b**) Scatter plot and linear regression fit of manual and algorithmic values. The black dashed line represents the ideal reference line *y* = *x*. The red solid line indicates the linear regression fit (*Y* = 0.9746*X* + 0.01538), with a coefficient of determination *R*^2^ = 0.9609.

The Bland–Altman analysis (Figure 6a) shows that the mean bias between the manual values and the algorithm-derived values is only 0.005532 Hz, with 95% limits of agreement ranging from −0.08133 Hz to 0.09239 Hz. The vast majority of data points covering the three different growth stages fall within this range. The scatter plot and linear regression analysis (Figure 6b) show that all data points cluster closely around the ideal reference line *y* = *x*. The linear regression equation is *Y* = 0.9746*X* + 0.01538, with a slope close to 1 and an intercept close to 0, and the coefficient of determination *R*^2^ = 0.9609.

**Figure 7:**
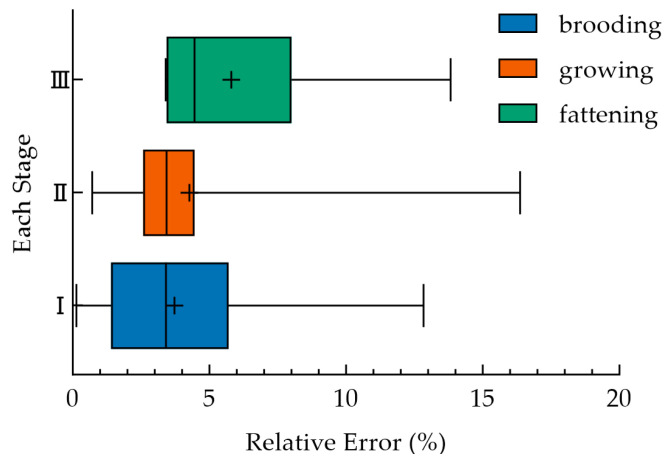
Boxplot of relative error in the whole-life-stage experiment. I, II, and III, respectively, represent the three stages of broiler: brooding, growing, and fattening. The “+” symbol in the boxplot denotes the mean value of relative error for each stage.

Figure 7 illustrates the distribution of the algorithm’s relative error across different growth stages. The median error for each stage was below 10%, and One-way analysis of variance confirmed that the error distribution exhibited significant inter-stage differences (*p* < 0.01). The median error and interquartile range were relatively large in the fattening stage, while the errors in the brooding and growing stages were concentrated in a lower range (median ≈ 3.5%). The results of the one-way analysis of variance (One-way ANOVA) were used to compare the differences in the means among the three groups. The results showed that *F* (2, 195) = 6.908, *p* = 0.0013, indicating significant differences among the three groups. Homogeneity of variance tests (Brown-Forsythe, *p* = 0.3122; Bartlett, *p* = 0.8843) confirmed that the data met the homogeneity of variance assumption. The effect size *η*^2^ = 0.06617, suggesting that the group factor explained 6.617% of the total variance. Tukey’s post hoc multiple comparisons showed that the mean relative error of the fattening group was significantly higher than that of the brooding group (*p* = 0.0028) and the growing group (*p* = 0.0084), while there was no significant difference between the brooding group and growing group (*p* = 0.6205).

#### 3.2.2. Experiment on Broiler RR Under Heat Stress

The heat stress experimental data were collected from broilers at 7, 8, and 9 days of age (brooding stage), 18, 19, and 20 days of age (growing stage), and 30, 31, and 32 days of age (fattening stage). For each of these nine days, the environmental temperature was increased by 2 °C, 4 °C, and 5 °C on the basis of the optimal temperature. On each experimental day and at each temperature increment, six non-repeated broilers were randomly selected from the same batch of broilers across five cages, and their RR was measured by both manual counting and the algorithm.

**Figure 8:**
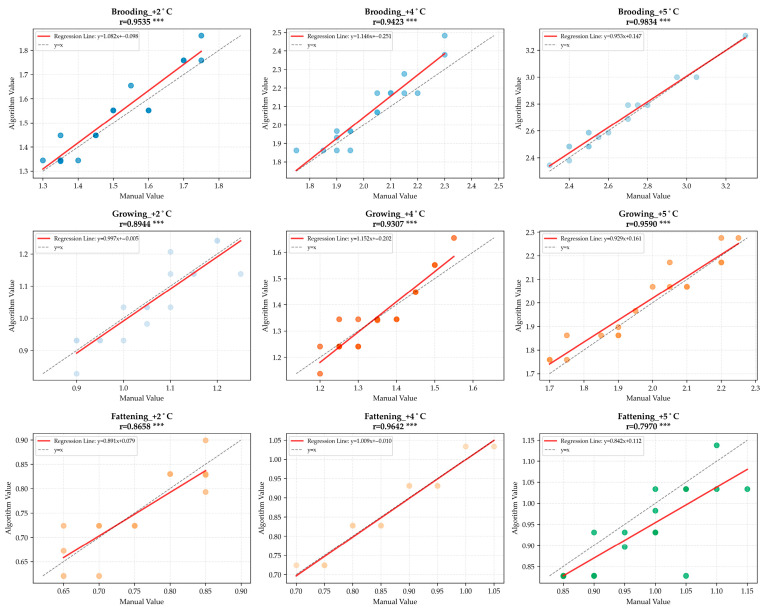
Scatter plot of the 9 groups in the heat stress experiment. All Pearson correlation coefficients were statistically significant (all *p* < 0.001, denoted by *** in the figure, where *** indicates *p* < 0.001). The specific *p*-values were as follows: for the brooding stage: +2 °C group *p* < 0.000001, +4 °C group *p* < 0.000001, +5 °C group *p* < 0.000001; for the growing stage: +2 °C group *p* = 0.000001, +4 °C group *p* < 0.000001, +5 °C group *p* < 0.000001; for the fattening stage: +2 °C group *p* = 0.000003, +4 °C group *p* < 0.000001, +5 °C group *p* = 0.000075.

Scatter analysis plots (Figure 8) show that most data points are closely distributed on both sides of the identity line (*y* = *x*) reference line in each subplot, with regression slopes close to 1. All groups have Pearson correlation coefficients (*r*) higher than 0.79, and most of them are above 0.93. All correlation tests yielded statistically significant *p*-values (*p* < 0.001). Specifically, the r values of each group in the brooding stage range from 0.942 to 0.983 (all *p* < 0.000001), those in the growing stage range from 0.894 to 0.959 (all *p* < 0.00001), and those in the fattening stage range from 0.797 to 0.964 (all *p* < 0.0001).

Table 3 summarizes the core evaluation metrics (MAE, MAPE, RMSE, and *R*^2^) from the two experiments. In the whole-life-stage experiment, the mean absolute error (MAE) was 0.036 Hz, the mean absolute percentage error (MAPE) was 4.461%, the root mean square error (RMSE) was 0.044 Hz, and *R*^2^ was 0.961. In the heat stress experiment, the MAE was 0.042 Hz, the MAPE was 3.270%, the RMSE was 0.055 Hz, and *R*^2^ was 0.928.

### 3.3. Benchmark Patterns of Broiler RR Across the Whole Life Stage

During the period from 4 to 36 days of age, the RR of healthy broilers in an optimal environment showed a significant linear downward trend with increasing age, presenting a distinct stage pattern: relatively high rates in the brooding stage, intermediate rates in the growing stage, and the lowest rates in the fattening stage.

**Figure 9:**
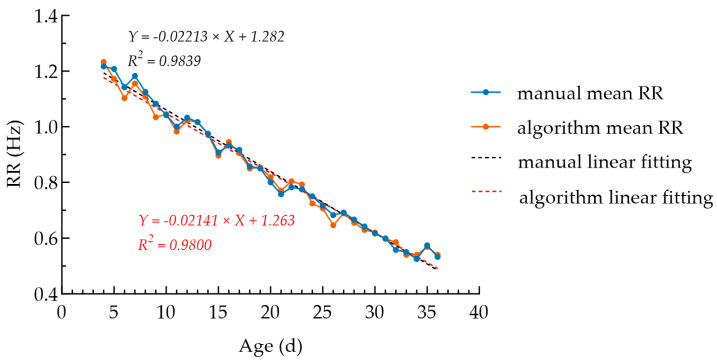
RR trend chart for broilers across the whole life stage. The mean standard deviation (SD) for manually counted RR was 0.12 Hz, while the mean SD for algorithmically detected RR was 0.13 Hz.

The two fitting lines for the manually measured mean and the algorithm-measured mean almost overlap (Figure 9). Their linear fitting equations are *Y* = −0.02213*X* + 1.282 and *Y* = −0.02141*X* + 1.263, respectively, with highly close slopes and goodness of fit *R*^2^ > 0.98 for both. Specifically, the slope for manually measured mean was −0.02213 Hz/day (95% CI: −0.02316 to −0.02109, *p* < 0.0001), and the slope for the algorithm-measured mean was −0.02141 Hz/day (95% CI: −0.02253 to −0.02029, *p* < 0.0001), indicating that the decreasing trend in RR with increasing age was statistically significant. For each additional day of age of broilers, the RR decreased by about 0.02 Hz (1.2 breaths per minute). The mean standard deviation (SD) of the manual counting and algorithm detection results were 0.12 Hz and 0.13 Hz, respectively.

**Figure 10:**
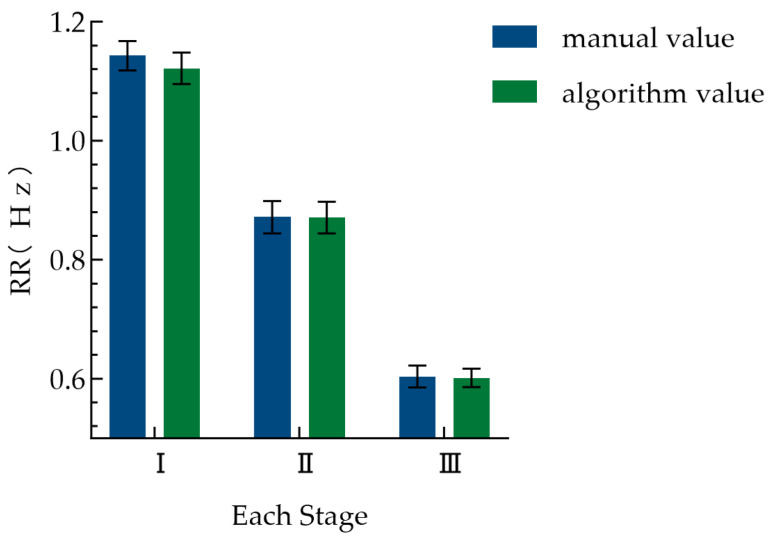
Bar chart of RR at different stages for broilers across the whole life stage. I, II, and III, respectively, represent the three stages of broiler: brooding, growing, and fattening. The error bars represent the standard error of the mean (SEM).

Stage-specific statistical results (Figure 10) show that the RR of the broilers was approximately 1.1 Hz in the brooding stage, decreased significantly to about 0.9 Hz in the growing stage, and further decreased to approximately 0.6 Hz in the fattening stage (the lowest level across the whole life stage). One-way ANOVA revealed that the differences in RR among the three stages were statistically significant (*F* (2, 63) = 183.5, *p* < 0.0001). Tukey’s post hoc multiple comparisons further indicated that pairwise differences between all stages were significant (all *p* < 0.0001). These results indicate that the decrease in RR between growth stages is statistically significant.

### 3.4. Analysis of Temperature Stress Responses Based on RR Changes

A temperature rise of +2 °C or higher induces a significant stepwise increase in RR, indicating a strong physiological response to thermal changes in the broilers. While this sharp increase in RR is typically indicative of heat stress, in the absence of additional physiological or behavioral validation, we interpret this phenomenon as a response to thermal challenge. The magnitude of RR changes shows a positive correlation with the thermal load intensity, and the response patterns display clear developmental stage specificity.

**Figure 11:**
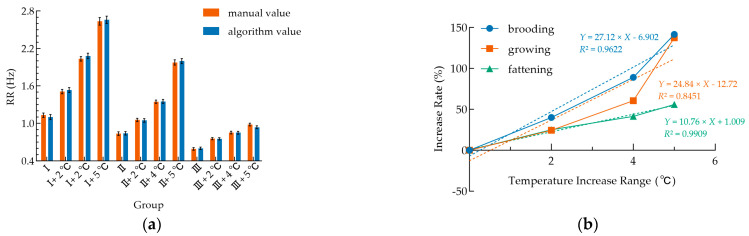
RR responses to temperature gradients and regression analysis of rate increases in broilers at different growth stages. (**a**) Bar charts showing the mean RR at different temperature gradients (control, +2 °C, +4 °C, +5 °C) across growth stages (I: brooding broilers, II: growing broilers, III: fattening broilers). Error bars represent SEM (all < 0.06 Hz); (**b**) Linear regression analysis of RR increases (relative to optimal temperature baseline) versus temperature elevation across growth stages. Fitted equations and *R*^2^ are labeled in the figure, quantifying the relationship between heat stress intensity and RR response.

The RR of broilers in all three growth stages showed an overall upward trend as the temperature increased from the control (optimal temperature) to +5 °C, with significant stage differences (Figure 11a). Broilers at the brooding and growing stages showed significant differences in RR among the temperature gradients, presenting a clear stepwise increase. In contrast, fattening broilers showed an upward trend but with no significant differences between gradients and a mild increase. Brooding broilers were the most sensitive to the temperature rise: at +4 °C, the RR increased sharply from approximately 1.1 Hz in the control group to about 2.1 Hz, and reached a peak of approximately 2.7 Hz at +5 °C, with an increase of more than 145%. For fattening broilers, the RR increased from approximately 0.6 Hz in the control group to about 0.9 Hz at +5 °C, with an increase of only about 50%.

Linear regression analysis (Figure 11b) showed that the RR increase (%) and the temperature increment (°C) presented a positive linear correlation at all three growth stages. The fattening broilers had the highest coefficient of determination (*R*^2^ = 0.9909), while the brooding broilers had the steepest slope in the regression equation (*Y* = 27.12*X* − 6.902).

## 4. Discussion

The Bland–Altman plot and regression plot (Figure 6) demonstrate excellent consistency between the algorithmic and manually counted RR values, with a mean bias of only 0.005532 Hz, narrow 95% limits of agreement, and a coefficient of determination *R*^2^ = 0.9609. These results confirm the favorable accuracy and reliability of the proposed algorithm in the whole-life-stage experiment. Scatter plots in Figure 8 further validate the strong linear correlations across all experimental groups, with Pearson correlation coefficients above 0.79 (most exceeding 0.93) and all correlations statistically significant (*p* < 0.001), indicating stable algorithm performance across growth stages and temperature gradients. As summarized in Table 3, the MAE and MAPE values for both experiments were below 0.05 Hz and 5%, respectively, with *R*^2^ close to 1, suggesting no obvious systematic overestimation or underestimation and high overall consistency with manual counting. To assess generalizability, we compared our algorithm with a previous study [23] that validated RR measurement only at room temperature or in a single growth stage, which reported an average accuracy (RRacc) of 92.19%. In contrast, the present study comprehensively verified the algorithm’s scenario adaptability using multi-stage and multi-gradient experiments. The proposed method addresses key challenges in broiler RR monitoring, including weak respiratory signals, small body size, and frequent occlusions, while reducing errors associated with manual counting, such as subjective judgment and visual fatigue. These results indicate that the algorithm provides a promising alternative to conventional manual counting under laboratory conditions, offering an efficient and objective tool for large-scale poultry heat stress studies and supporting the development of automated monitoring systems.

Notably, as shown in Figure 7, the algorithm’s error distribution showed significant stage differences, with a relatively larger median error and interquartile range in the fattening stage. This is mainly because the absolute RR of fattening broilers is low, and the same absolute error leads to a larger relative error. This phenomenon can be explained by the synergy of the physiological development of broilers, the characteristics of RR signals, and the detection principle of the algorithm. In the fattening stage, the rapid weight gain and increased thoracic fat deposition of broilers may have significantly changed their breathing patterns, showing shallower respiration and lower RR. Moreover, the activity of broilers decreases in the late fattening stage, making RR signals weaker and gentler. According to the detection principle of the algorithm, the RR was extracted based on the visual motion features of the thoracodorsal region and tail of broilers. When RR signals had a small amplitude and low frequency, minor detection deviations, such as interframe motion misjudgment, are amplified into larger relative errors under a low-frequency base. In addition, the large difference in obesity among individuals may further increase the variability of breathing patterns, eventually leading to a more discrete error distribution at this stage. In contrast, the errors in the brooding and growing stages were concentrated in a lower range, and the algorithm showed better stability. This may be mainly attributed to the vigorous metabolism and high RR of broilers at these stages, with clear motion amplitude in the thoracodorsal region and tail. The temporal characteristics of the RR signals were more significant, so the algorithm was more robust in identifying such high-frequency and strong signals, with smaller absolute detection errors. Furthermore, the higher activity of broilers in the brooding and growing stages made it easier to distinguish RR signals from environmental noise, further reducing the probability of misjudgment. These findings suggest that the signal enhancement and motion discrimination modules of the algorithm can be optimized according to the physiological characteristics of fattening broilers, so as to improve the universality of the algorithm across the whole growth stage.

This study systematically established the baseline characteristics of the RR in healthy white-feathered broilers under optimal conditions. As shown in Figure 9, the RR of healthy broilers decreased linearly with increasing age, from approximately 1.2 Hz at the brooding stage to about 0.5 Hz at the fattening stage, which is similar to the research results of Nascimento et al. [28], who conducted weekly RR studies on two broiler breeds. As shown in Figure 10, the RR of the broilers was approximately 1.1 Hz in the brooding stage, decreased significantly to about 0.9 Hz in the growing stage, and further decreased to approximately 0.6 Hz in the fattening stage, presenting the RR patterns across different growth stages. This downward trend is mainly related to the growth and development characteristics of broilers: with the increase in age, the metabolic rate per unit body weight decreases relatively, and the thermoregulatory system becomes increasingly improved, leading to a gradual decrease in the RR required to maintain physiological homeostasis. The highly overlapping fitting curves of manual measurement and algorithm measurement further verify the accuracy of the algorithm to replace manual measurement on the whole-life-stage scale. The establishment of this preliminary baseline RR curve provides a preliminary reference for the subsequent identification of respiratory abnormalities deviating from the normal developmental trajectory and lays a foundation for the respiratory health assessment of broilers.

In terms of heat stress response. As shown in Figure 11a, a temperature rise of 2 °C or more triggers physiological compensatory responses in the broiler RR, which increases gradually with temperature. This finding aligns with Brugaletta et al. [29], who reported that broilers accelerate RR to enhance evaporative cooling as a key compensatory strategy for thermoregulatory homeostasis. Meanwhile, broiler heat stress responses show clear growth stage specificity: brooding broilers are the most sensitive to temperature increases, while fattening broilers exhibit the most stable linear response pattern. This difference arises because the thermoregulatory system of the brooding broilers is underdeveloped—with a high metabolic rate and weak heat dissipation—leading to more intense respiratory compensation, whereas fattening broilers have mature thermoregulatory mechanisms and a gentler response. Notably, the RR in this study only acts as an early physiological compensatory indicator and cannot independently determine the severity of heat stress or represent a validated animal welfare outcome. This quantitative relationship confirms that the RR can be used as a sensitive early warning indicator for thermal challenge in brooding and growing broilers. Though fattening broilers show the strongest linear response, their mild absolute RR changes mean this single indicator is insufficient for direct heat stress judgment, requiring a combination with other physiological indicators for a comprehensive assessment. These findings provide core data support for stage-specific environmental regulation and refined management of broilers across growth stages.

Despite the above progress, this study still has certain limitations. First, all experiments were carried out under controlled laboratory conditions to reduce the influence of environmental fluctuations on experimental data, and they have not been systematically validated in the complex scenarios of actual commercial farms. In real farming environments, factors such as lighting variation, dust interference, and high stocking density may affect infrared thermal imaging quality and algorithm performance, which will require further evaluation in future field studies. Second, the existing algorithm has not achieved staged optimization in signal enhancement and visual feature extraction for the physiological differences in broilers at different growth stages, so there is still room for improvement in the monitoring accuracy and heat stress judgment performance for the fattening broilers.

Future research will focus on the following aspects: (1) Optimizing the video magnification parameters and visual feature extraction strategies according to the physiological characteristics of the fattening broilers, to improve the applicability and robustness of the algorithm across the whole life stage. (2) Introducing coupled modeling of environmental factors such as temperature and humidity in real farming scenarios, to further improve the adaptability of the model to complex breeding environments. (3) Extending the research objects to multiple broiler breeds and other livestock to systematically evaluate the universality of the method.

## 5. Conclusions

This study integrated video magnification and infrared thermal imaging technologies to explore a non-contact method for measuring the respiratory rate (RR) of caged broilers based on the phase-based video magnification (PBVM) algorithm. Under controlled laboratory conditions, the method demonstrated favorable measurement performance in both whole-life-stage dynamic monitoring and graded heat stress experiments. In the whole-life-stage experiment with healthy broilers, the MAE was 0.036 Hz and the *R*^2^ was 0.961. In the heat stress experiment, the MAE was 0.042 Hz and the *R*^2^ reached 0.928. Meanwhile, this study systematically clarified two core physiological patterns in broilers. First, the healthy broilers’ RR declined linearly with age under optimal conditions. Second, the RR increased stepwise with a temperature rise of 2 °C or above, with distinct stage-specific response characteristics.

At the methodological level, this study provides a feasible technical route and methodological reference for the non-invasive and continuous monitoring of poultry physiological indicators. At the research level, the established whole-life-stage RR baseline for healthy broilers offers a preliminary reference for respiratory abnormality recognition and the health assessment of broilers, and the revealed stage-dependent response patterns of the RR under heat stress provide data support for staged environmental regulation and refined management of broilers. It should be noted that all experiments in this study were conducted under controlled laboratory conditions with a limited sample size. External validation is still required in commercial farm scenarios before large-scale application. The above results provide a preliminary basis for broiler health monitoring, animal welfare improvement, and the practical application of precision livestock farming.

## Figures and Tables

**Table 1:** Video shooting parameters of infrared thermal imager.

Parameter Type	Parameter Value
Placement Height	0.6 m~0.9 m
Distance from Broiler Cage	0.1 m~0.5 m
Video Frequency	30 frames per second
Video Resolution	640 × 480
Color Model	Rainbow
Emissivity(ε)	0.95

**Table 2:** Hardware and software configuration details for the experiment.

Hardware and Software Name	Configuration Details
PyTorch	2.1.2
Python	3.10
CUDA	11.8
CPU	15 vCPU Intel(R) Xeon(R) Platinum 8358P CPU @ 2.60 GHz, Intel Corporation, Santa Clara, CA, USA
GPU	NVIDIA RTX 4090(24 GB), NVIDIA Corporation, Santa Clara, CA, USA
Memory	90 GB

**Table 3:** Evaluation results of broiler RR measurement in two experiments.

Experimental Type	MAE	MAPE	RMSE	*R* ^2^
Whole-Life-Stage Experiment	0.036 Hz	4.461%	0.044 Hz	0.961
Heat Stress Experiment	0.042 Hz	3.270%	0.055 Hz	0.928

## Data Availability

The data presented in this study are available on request from the corresponding author.
